# Mydgf promotes Cardiomyocyte proliferation and Neonatal Heart regeneration

**DOI:** 10.7150/thno.44281

**Published:** 2020-07-11

**Authors:** Yuyao Wang, Yan Li, Jie Feng, Weijing Liu, Yandong Li, Jun Liu, Qianqian Yin, Hong Lian, Lihui Liu, Yu Nie

**Affiliations:** 1 Department of Biochemistry and Molecular Biology, Shanxi Medical University, Taiyuan 030001, China.; 2 State Key Laboratory of Cardiovascular Disease, Fuwai Hospital, National Center for Cardiovascular Disease, Chinese Academy of Medical Sciences and Peking Union Medical College, Beijing 100037, China.

**Keywords:** Mydgf, cardiomyocyte proliferation, heart regeneration, c-Myc, FoxM1

## Abstract

Myeloid-derived growth factor (Mydgf), a paracrine protein secreted by bone marrow-derived monocytes and macrophages, was found to protect against cardiac injury following myocardial infarction (MI) in adult mice. We speculated that Mydgf might improve heart function *via* myocardial regeneration, which is essential for discovering the target to reverse heart failure.

**Methods:** Two genetic mouse lines were used: global Mydgf knockout (*Mydgf-KO*) and *Mydgf-EGFP* mice. Two models of cardiac injury, apical resection was performed in neonatal and MI was performed in adult mice. Quantitative reverse transcription-polymerase chain reaction, western blot and flow cytometry were performed to study the protein expression. Immunofluorescence was performed to detect the proliferation of cardiomyocytes. Heart regeneration and cardiac function were evaluated by Masson's staining and echocardiography, respectively. RNA sequencing was employed to identify the key involved in Mydgf-induced cardiomyocyte proliferation. Mydgf recombinant protein injection was performed as a therapy for cardiac repair post MI in adult mice.

**Results:** Mydgf expression could be significantly induced in neonatal mouse hearts after cardiac injury. Unexpectedly, we found that Mydgf was predominantly expressed by endothelial cells rather than macrophages in injured neonatal hearts. Mydgf deficiency impeded neonatal heart regeneration and injury-induced cardiomyocyte proliferation. Mydgf recombinant protein promoted primary mouse cardiomyocyte proliferation. Employing RNA sequencing and functional verification, we demonstrated that c-Myc/FoxM1 pathway mediated Mydgf-induced cardiomyocyte expansion. Mydgf recombinant protein improved cardiac function in adult mice after MI injury with inducing cardiomyocyte proliferation.

**Conclusion:** Mydgf promotes cardiomyocyte proliferation by activating c-Myc/FoxM1 pathway and improves heart regeneration both in neonatal and adult mice after cardiac injury, providing a potential target to reverse cardiac remodeling and heart failure.

## Introduction

Heart disease continues to be the leading public health problem worldwide 1-4. The adult mammalian heart is incapable of meaningful self-recovery after injury owing to losing cardiomyocyte (CM) proliferative capacity, which is the basis for poor regenerative capacity and defective repair of the adult heart in response to cardiac injury, such as myocardial infarction (MI) and heart failure 5-7. Unlike adult, neonatal mammalian heart is able to regenerate after various types of injury 8, but this regenerative process seems to be limited to the first postnatal week 9, 10, which coincides with CM exiting from the cell cycle 11-13, CM cell-cycle withdrawal during mammalian postnatal development is tightly coupled with CM polyploidization, an increase in nuclear DNA content 14. It has been demonstrated that troponin I3 kinase levels 15 or insulin-like growth factor 1 16 could change the percentage of polyploid CM nuclei, which altered heart regeneration 17.

Myeloid-derived growth factor (Mydgf), a paracrine protein produced by bone marrow-derived mononuclear macrophages (MΦs), could reduce scar size and improve heart function after MI 18. Recently, it has been demonstrated that Mydgf-based biologically active recombinant human Mydgf could mediate endothelial cell (EC) proliferation *via* the MAPK-STAT3 signaling pathways 19. However, whether Mydgf could promote CM proliferation and heart regeneration remains unknown, which is the promising approach to reverse cardiac remodeling and hear failure. Mydgf was predominantly expressed by Ly6C^high^ monocytes, Ly6C^low^ monocytes or MΦs, T cells, B cells, ECs and CMs 18. The source of Mydgf in myocardium should be estimated after cardiac injury, which will provide the target cells to improve cardiac regeneration.

In this study, we found that Mydgf expression could be induced significantly in neonatal mouse hearts after cardiac injury. Mydgf deficiency impeded CM proliferation and heart regeneration. Mydgf recombinant protein administration could promote CM proliferation *in vitro* and *in vivo*. RNA sequencing identified c-Myc/FoxM1 pathway mediated the pro-proliferative role of Mydgf. Mydgf treatment promoted CM proliferation and heart regeneration in adult mice after MI, suggesting that Mydgf might be an effective regenerative factor for injured heart recovery.

## Materials and Methods

### Mice

The *C57BL/6J* mice used for wild-type (WT) studies were purchased from Vital River Laboratory Animal Technology Co. Ltd. (Beijing, China).

*Mydgf-flox* mouse line (*Cre-B6; 129-Mydgf^tm1(flox)Smoc^; Cat. NO. NM-CKO-00021*) was constructed from Shanghai Model Organisms Center, Inc (Shanghai, China). To generate Mydgf null mice, we crossed the *Mydgf-flox* mice to *EIIa-Cre* mice which express Cre ubiquitously from the EIIa promoter. Heterozygous Mydgf-targeted, *EIIa-Cre* transgene-positive mice were crossed to WT mice to generate heterozygous Mydgf-deleted, Cre transgene-negative (*Mydgf^+/-^*) mice, which were used to produce *Mydgf-KO* mice.

*Mydgf-EGFP* mice were generated by Beijing Biocytogen Co., Ltd. (Beijing, China). For generating *Mydgf-EGFP* mice, sgRNAs were designed to target the region near Stop codon. For the targeting site, candidate guide RNAs were designed by the CRISPR design tool (http://www.sanger.ac.uk/htgt/wge/). Guide RNAs were screened for on-target activity use UCATM. UCATM (Universal CRISPR Activity Assay), a sgRNA activity detection system developed by Biocytogen, was simpler and more sensitive than MSDase assay. The following primers were used to amplify the 3'-external probe (374 bp): ACCATTGAGAGAAGCCACGATCTGC (forward primer) and TGCTGGATGATAGGTGTGTGCCAAC (reverse primer). For the internal probe (526 bp), the following primers were used: TCGTGACCACCCTGACCTACG (forward primer) and TCGTCCATGCCGAGAGTGATCC (reverse primer).

Mice were sacrificed using cervical dislocation before collecting hearts. For echocardiography, isoflurane (2.5%) was used to anaesthetize the mice prior to echocardiography.

All animal procedures were approved by the Institutional Animal Care and Use Committee (IACUC), Fuwai Hospital, Chinese Academy of Medical Sciences (Beijing, China) and performed in accordance with the Guide for the Care and Use of Laboratory Animals published by the US National Institutes of Health (NIH).

### Mydgf antibody

We commissioned Absin Bioscience Inc (China Shanghai) to produce Mydgf antibody. We raised a rabbit polyclonal antibody against a peptide sequence in the secreted portion of Mydgf (c-VSEPTTVPFDVRPGG). We purified the antibody by affinity purification. We documented the specificity of the antibody by immunoblot analysis.

### Mydgf recombinant protein

Mydgf recombinant protein was purchased from Protein Specialists (CYT-1040). For cardiomyocyte (CM) treated with Mydgf recombinant protein experiment, 50 ng/ml concentration was used. We explored whether Mydgf promoted cardiomyocyte proliferation *in vivo* through apical intramyocardial microinjection of Mydgf recombinant protein (1 μg/mouse) in neonatal mice. For adult MI, we microinjected Mydgf recombinant protein (5 μg/mouse) into 6-8 weeks mice myocardium after MI followed by a continuous tail vein injection for 7 days (10 µg/d).

### Apical resection

The heart apical resection (AR) model of neonatal mouse was operated as described previously 20, 21. Briefly, neonatal mouse at postnatal day 1 (P1) was anesthetized by hypothermia on an ice bed for 1.5 minutes. A 1 cm incision was made at the fourth intercostal space and steady pressure was applied to exteriorize the heart out of the chest. The left ventricle apex was amputated using iridectomy scissors. Subsequently, the chest and skin incisions were sewn up with an 8/0 non-absorbable silk suture. The neonates were warmed under a heat bed for several minutes until the pups came to life. In the sham controls, we performed the same procedures without truncating the heart apex.

### Myocardial infarction

For the murine myocardial infarction (MI) at adult stage (6-8 weeks) induced by ligating the left anterior descending (LAD) coronary artery as previously described. The adult mice were anesthetized by intraperitoneal injection of pentobarbital sodium (40 mg/kg; Solarbio, Beijing, China). After the chest was opened at the fourth intercostal space, and a suture was placed around the LAD coronary artery to inflict myocardial ischemia on the left ventricle. Then, the incision was sewed up with a 6/0 non-absorbable silk suture, and the mice were warmed under a heat bed until they came to life. In the sham controls, we performed the same procedures without suturing the LAD coronary artery.

### Echocardiography

The left ventricle systolic function was analyzed by echocardiography at 21 days after AR or MI using a digital ultrasound system (Vevo2100 Imaging System, Visual Sonics, Toronto, Canada). Isoflurane (2.5%) was used to anaesthetize the mice prior to echocardiography. The systolic function was measured by left ventricle ejection fraction (LVEF).

### Histology

The harvested hearts were fixed in 4% paraformaldehyde at room temperature for 48 hours, then dehydrated in an ethanol and xylene series, and finally paraffin-embedded. For the AR model, hearts were continuously sectioned at 5 μm thickness until the injured section was observed. For the MI model, hearts were continuously sectioned at 5 μm thickness from the ligation site to the apex at an interval of 200 μm between each section; five sections were collected from each heart. Masson's staining was performed using standard procedures 22.

### Cell sorting

We isolated the mouse hearts at 1 day after AR using the Neonatal Heart Dissociation Kit in combination with gentleMACS^TM^ and Octo Dissociator (Miltenyi BioTec, Teterow, Germany) according to the manufacturer's instructions. Then we used the Neonatal Cardiomyocyte Isolation Kit (130100825, Miltenyi BioTec, Teterow, Germany) to isolate mouse CMs by depletion of non-target cells. Nontarget cells were directly magnetically labeled with a cocktail of monoclonal antibodies conjugated with MACS MicroBeads. The magnetically labeled non-target cells were depleted by retaining them within a MACS (MS) column in the magnetic field of a MACS Separator, while the unlabeled CMs passed through the column. As for macrophages (MΦs) and endothelial cells (ECs), we incubated the target cells for 30 minutes at 4°C with the following antibodies: CD45-Percp (1:100, BD Biosciences, 557235), CD11b-PE (1:200, BD Biosciences, 557397), CD31-APC (1:200, Biolegend, 102419). After washing, we sorted the cells by flow cytometry on a FACS Aria Flow Cytometer (BD Biosciences, San Jose, CA, USA). We identified MΦs as CD45^+^CD11b^+^, ECs as CD31^+^.

### Cardiomyocyte isolation, culture, and siRNA transfection

For Mydgf recombinant protein experiments, CMs were isolated from hearts of WT mice at P1 using the Neonatal Heart Dissociation Kit in combination with gentleMACS^TM^ and Octo Dissociator (Miltenyi BioTec, Teterow, Germany) according to the manufacturer's instructions. CMs were plated into DMEM/F12 medium supplemented with 10% FBS at 37 °C and 5% CO_2_. Then CMs were treated with Mydgf recombinant protein and harvested 16 hours later, and analyzed by immunofluorescence and RNA sequencing (RNA-Seq). For inhibition of Akt, CMs were treated with Akt inhibitor LY294002 (Sigma, USA). For siRNA experiments, CMs were respectively transfected with siRNA-Akt, siRNA-c-Myc, siRNA-FoxM1 or negative control (NC) using Lipofectamine 3000 (Invitrogen, Waltham, USA) transfection reagent as previously described 22, harvested 48 hours later, and analyzed by western blot, immunofluorescence, or quantitative real-time Polymerase Chain Reaction (qRT-PCR). The siRNA-Akt sequences were: 5′-GCUACUUCCUCCUCAAGAATT-3′, 5′-UUCUUGAGGAGGAAGUAGCTT-3′, the siRNA-c-Myc sequences were: 5′-CCGUACAAGCCCUAUUUCAUTT-3′, 5′-AUGAAAUAGGGCUGUACGGTT-3′, the siRNA-FoxM1 sequences were: 5′-GCAAAUUUCCAGCCGGAAUTT-3′, 5′-AUUCCGGCUGGAAAUUUGCTT-3′, and the NC sequences were: 5′-UUCUCCGAACGUGUCACGUTT-3′, 5′-ACGUGACACGUUCGGAGAATT-3′.

### RNA sequencing

After treated with PBS or Mydgf recombinant protein for 16 hours, neonatal primary CMs were harvested, and the total RNA was extracted by RNeasy mini kit (QIAGEN). The quantity and quality of total RNA were measured by Nanodrop 2000 and Agilent Bioanalyzer 2100, respectively. The total RNA of 1 μg with a RIN value no less than 8.0 for each sample was used for library preparation. cDNA libraries were prepared and sequenced on an Illumina Miseq using the paired-end run methodology at Novogene. The raw reads were generated, and quality control determination was performed using standard protocols. RNA-Seq reads were aligned to the mouse genome by HISAT2 (Version 2.0.5), and gene expression level was quantified as fragments per kilo base of exon per million fragments (FPKM) using featureCounts (Version 1.5.0-p3). DESeq2 (Version 1.16.1) was used to determine genes differentially expressed between Mydgf recombinant protein and PBS group. Genes which FPKM > 1 with the fold change > 2 and FDR < 5% were considered to be expressed differently as described previously 23. KEGG (Kyoto encyclopedia of genes and genomes) enrichment analysis was conducted using Gene Group Functional Profiling. Variables with adjusted *P* values of < 0.05 were considered significant enrichment. The accession number of the RNA sequencing data reported in this paper is Sequence Read Archive (SRA): PRJNA600826.

### Immunofluorescence

Deparaffinizing, antigen retrieval, and blocking of non-specific background were performed as described previously 22 and sections were incubated with primary antibodies overnight at 4°C. After washing three times with PBS, and incubated with fluorescence-labeled secondary antibodies for 1 hour at room temperature in the dark. Slices were washed three times in PBS, and then counterstained with DAPI (Sigma-Aldrich, St Louis, MO, USA). The primary antibodies were as follows: anti-sarcomeric alpha actinin (1:200; ab9465, Abcam, UK), anti-phosphorylated Histone 3 (1:1000; 3377, Millipore, USA), anti-Ki67 (1:200; ab16667, Abcam, UK), anti-Aurkb (1:100; A5102, Sigma, USA), anti-CDH5 (1:100; ab33168, Abcam, UK) and anti-Vimentin (1:100; ab92547, Abcam, UK). The secondary antibodies were from Invitrogen: anti-rabbit Alexa Fluor 488-conjugated (1:400; A21206, Invitrogen, USA), anti-mouse Alexa Fluor 594-conjugated (1:400; A21203, Invitrogen, USA). Fluorescence was observed under a ZEISS LSM800 confocal laser scanning microscope (Carl Zeiss, Inc., Jena, Germany). We used High Content Screening System (Opera Phenix) to count cell proliferation ratio.

### Ploidy measurement on mouse heart sections by microscopy

Non-Langendorff perfusion method was used to isolate WT/*Mydgf-KO* mouse hearts at P14 as described previously 23. Cells were further washed with PBS three times, deposited on slices, and then allowed to dry out completely. The sections were incubated with anti-sarcomeric alpha actinin (1:200; ab9465, Abcam, UK) overnight at 4°C. After washing three times with PBS, these slices were incubated with anti-mouse Alexa Fluor 594-conjugated (1:400; A21203, Invitrogen, USA) for 1 hour at room temperature in the dark. Slices were washed three times in PBS, counterstained with DAPI (Sigma-Aldrich, St Louis, MO, USA). Fluorescence was observed under a ZEISS LSM800 confocal laser scanning microscope (Carl Zeiss, Inc., Jena, Germany). The ploidy of CM nuclei was determined by normalizing DAPI intensity of CM nuclei to that of non-CM nuclei in the same field. Non-CMs were assumed diploid (2N). In details, to demarcate nuclear boundaries, images of the DAPI signal were processed in Image J. After nuclear boundaries were identified, the integrated pixel density of individual nuclei in CMs and non-CMs were measured manually. All raw integrated pixel density values per each nuclei were organized in an excel spreadsheet. CM nuclei values were normalized against the average value observed in non-CM nuclei in the same imaging field. Normalized readings were then organized into histograms. At least 400 CMs per each sample were analyzed.

### Western blot

Hearts and CMs were harvested and incubated in RIPA lysis buffer with 1 mM phenylmethylsulfonyl fluoride (PMSF) (Beyotime Institute of Biotechnology, Beijing, China). After homogenization and incubation at 4 °C for 40 minutes, all protein samples were mixed at 1:4 with 4 × SDS loading buffer and 1:10 with 10 × SDS loading buffer for 10 minutes at 70 °C. Subsequently, 10 μg of total protein were loaded onto 10% sodium dodecyl sulfate-polyacrylamide gel electrophoresis and transferred to a nitrocellulose membrane. After blocking, the membranes were incubated at 4 °C overnight with the following primary antibodies: anti-Mydgf (1:1000), anti-Akt (1:1000; 4691, Cell Signaling Technology, USA), anti-phosphorylated Akt (1:1000; 4060, Cell Signaling Technology, USA), anti-c-Myc (1:1000; 18583, Cell Signaling Technology, USA), anti-FoxM1 (1:1000; PA5-71455, Thermo Fisher, USA), anti-Cyclin B1 (1:1000; 12231, Cell Signaling Technology, USA), anti-Cyclin D1 (1:1000; 55506, Cell Signaling Technology, USA), anti-CDK1 (1:1000; 9116, Cell Signaling Technology, USA), anti-CDK6 (1:1000; 13331, Cell Signaling Technology, USA), anti-p27 (1:1000; 3686, Cell Signaling Technology, USA), anti-STAT3 (1:1000; 12640, Cell Signaling Technology, USA), anti-phosphorylated STAT3 (S727) (1:1000; 34911, Cell Signaling Technology, USA) and anti-phosphorylated STAT3 (Y705) (1:1000; 9145, Cell Signaling Technology, USA). The membranes were washed with Tris-buffered saline/0.1% Tween 20 (TBST) and incubated with secondary antibodies for 1 hour at room temperature. Signals were detected using Pierce™ ECL Western Blot Substrate (Thermo Fisher Scientific, Waltham, USA).

### qRT-PCR

To quantify gene expression, the total RNA was extracted from heart samples or CMs using TRIzol reagent (Invitrogen) and then converted to cDNA using the PrimeScript RT Master Mix (Takara, RR036A). qRT-PCR was performed in triplicate using SYBR Green qPCR master mix (Applied Biosystems, USA) and amplified on a Vii7 Real-Time PCR System. All primers for the reactions were listed below.

Mydgf forward: 5′-TCGTGCATTCGTTCTCCC-3′, reverse: 5′-GCTCGTTGGTCCCTCCTT-3′; c-Myc forward: 5′-AGAGCTCCTCGAGCTGTTTG-3′, reverse: 5′-TCCTCGTCGCAGATGAAATA-3′; FoxM1 forward: 5′-AGCGTTAAGCAGGAACTGGA-3′, reverse: 5′-GGAAGTGGTCCTCAATCCAA-3′; Ki67 forward: 5′-CAGTACTCGGAATGCAGCAA-3′, reverse: 5′-CAGTCTTCAGGGGCTCTGTC-3′; Cyclin B1 forward: 5′-CTTGAACATGTTAGAGAAGAGAAGC-3′, reverse: 5′- TCGGGCTTGGAGAGGGATTA-3′; CDK1 forward: 5′-GGACGAGAACGGCTTGGATT-3′, reverse: 5′-ACACGATCTTCCCCTACGAC-3′; Gapdh forward: 5′-GCAGTGGCAAAGTGGAGATTG-3′, reverse: 5′-AGAGATGATGACCCTTTTGGCTCC-3′.

### Statistical analysis

All results were analyzed using GraphPad Prism (version 7.0, GraphPad Software). All data were expressed as the mean ± standard error of the mean (S.E.M.). Differences between groups were evaluated using an unpaired Student's *t*-test. When two groups of samples were compared for iterating parameters or when more than two groups of samples were compared, one-way ANOVA and two-way ANOVA were used. The Bonferroni's multiple comparisons test was used for the post hoc analysis of ANOVA results. A Kaplan-Meier curve was used to illustrate cumulative survival after MI and differences in cumulative survival were assessed using the log-rank test. Significance levels were indicated as suggested by Prism Software: *P* < 0.05 was considered statistically significant.

## Results

### Mydgf is induced during neonatal heart regeneration

**Figure 1:**
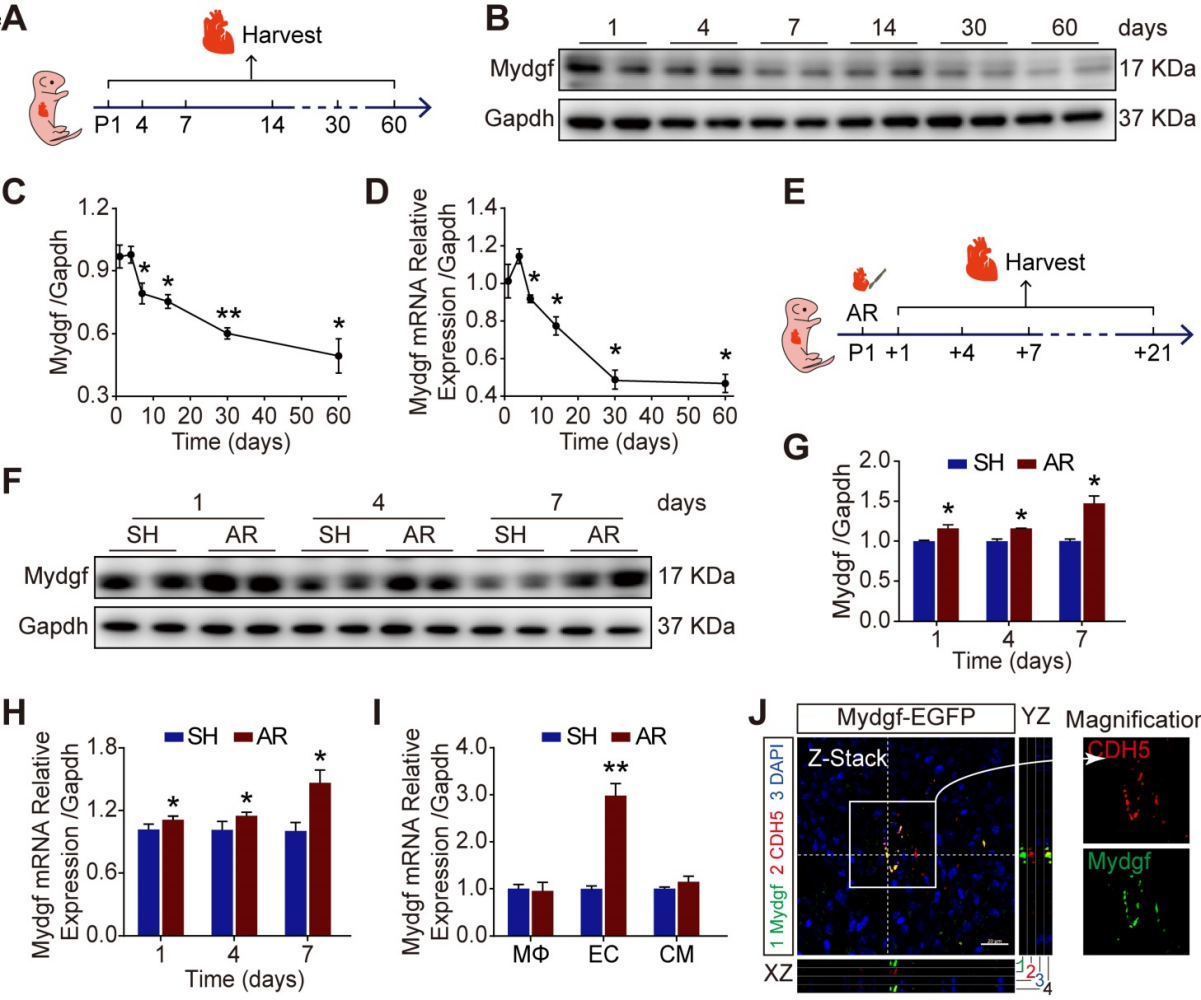
** Mydgf is induced during neonatal heart regeneration.** (**A**) Schematic diagram showed the experimental design for B-D. (**B-C**) Western blot analysis of Mydgf expression in wild-type (WT) mouse heart at different ages. Statistical analysis revealed that the expression of Mydgf decreased with age (n = 3 per group). (**D**) qRT-PCR analysis of Mydgf expression in WT mouse heart at different ages. Statistical analysis revealed that the expression of Mydgf decreased with age (n = 3 per group). *P < 0.05 and **P < 0.01 compared to postnatal day 1 (P1) by one-way ANOVA with Bonferroni's multiple comparisons test (C, D). (**E**) Schematic diagram showed the experimental design for F-M. (**F-G**) Western blot analysis of Mydgf in WT mouse hearts harvested at 1, 4, 7 days after apical resection (AR). Statistical analysis revealed that the expression of Mydgf was upregulated post neonatal heart injury (n = 3 per group). (**H**) qRT-PCR analysis of Mydgf in WT mouse hearts harvested at 1, 4, 7 days after AR. Statistical analysis revealed that the expression of Mydgf was upregulated post neonatal heart injury (n = 3 per group). (**I**) qRT-PCR analysis of Mydgf expression in three cell populations sorted by flow cytometry at 1 day after AR. Statistical analysis revealed that the expression of Mydgf was upregulated in endothelial cells post neonatal heart injury (n = 3 per group). *P < 0.05 and **P < 0.01 compared to SH at corresponding time-point by Student's *t*-test (G, H and I). (**J**) Mydgf and CDH5 (endothelial cell marker) were co-located by staining for EGFP (green), CDH5 (red), and nuclei (blue) at 7 days after AR in *Mydgf-EGFP* mice. Scale bars, 20 µm. Values were presented as the mean ± S.E.M.

We detected the expression of Mydgf by qRT-PCR and western blot in wild-type (WT) mouse hearts during postnatal development. We found that Mydgf was slightly increased at postnatal day 4 (P4) (n = 3 per group) and then gradually decreased with age (Figure 1A-D). To investigate whether Mydgf was involved in neonatal heart regeneration, we performed apical resection (AR) on P1 heart and measured the expression of Mydgf at different time points after resection (Figure 1E). Western blot and qRT-PCR revealed a significant increase of Mydgf expression at 1, 4, 7, 14 and 21 days post resection (dpr) (n = 3 per group) (Figure 1F-H, Supplementary Figure 1A-B), which coincided with the cardiomyocyte (CM) proliferation during neonatal heart regeneration 11.

To identify the cell types responsible for Mydgf production during heart regeneration, we sorted macrophages (MΦs), CMs and endothelial cells (ECs) using flow cytometry after AR and validated the purity of three cell populations by qRT-PCR (Supplementary Figure 1C-F). We found that Mydgf was predominantly expressed by ECs rather than MΦs 18 in the heart at 1 dpr by qRT-PCR (Figure 1I). In addition, we constructed *Mydgf-EGFP* mice, containing linker-EGFP and 2 homology arms of left (1302 bp) and right (1765 bp), to investigate the cells synthesized Mydgf. Immunostaining revealed that EGFP co-localized with CDH5^+^ cells, the marker of ECs at 7 dpr, indicating that the ECs are the predominant cell type after neonatal heart injury (Figure 1J).

### Mydgf is necessary for heart regeneration and CM proliferation

To investigate the role of Mydgf for heart regeneration, we introduced *Mydgf-knockout* (*Mydgf-KO*) mice and confirmed that Mydgf was effectively deleted by qRT-PCR (Supplementary Figure 2A). Echocardiography showed that *Mydgf-KO* mice kept normal heart function till P21 compared with WT littermates (Supplementary Figure 2B-2C).

**Figure 2:**
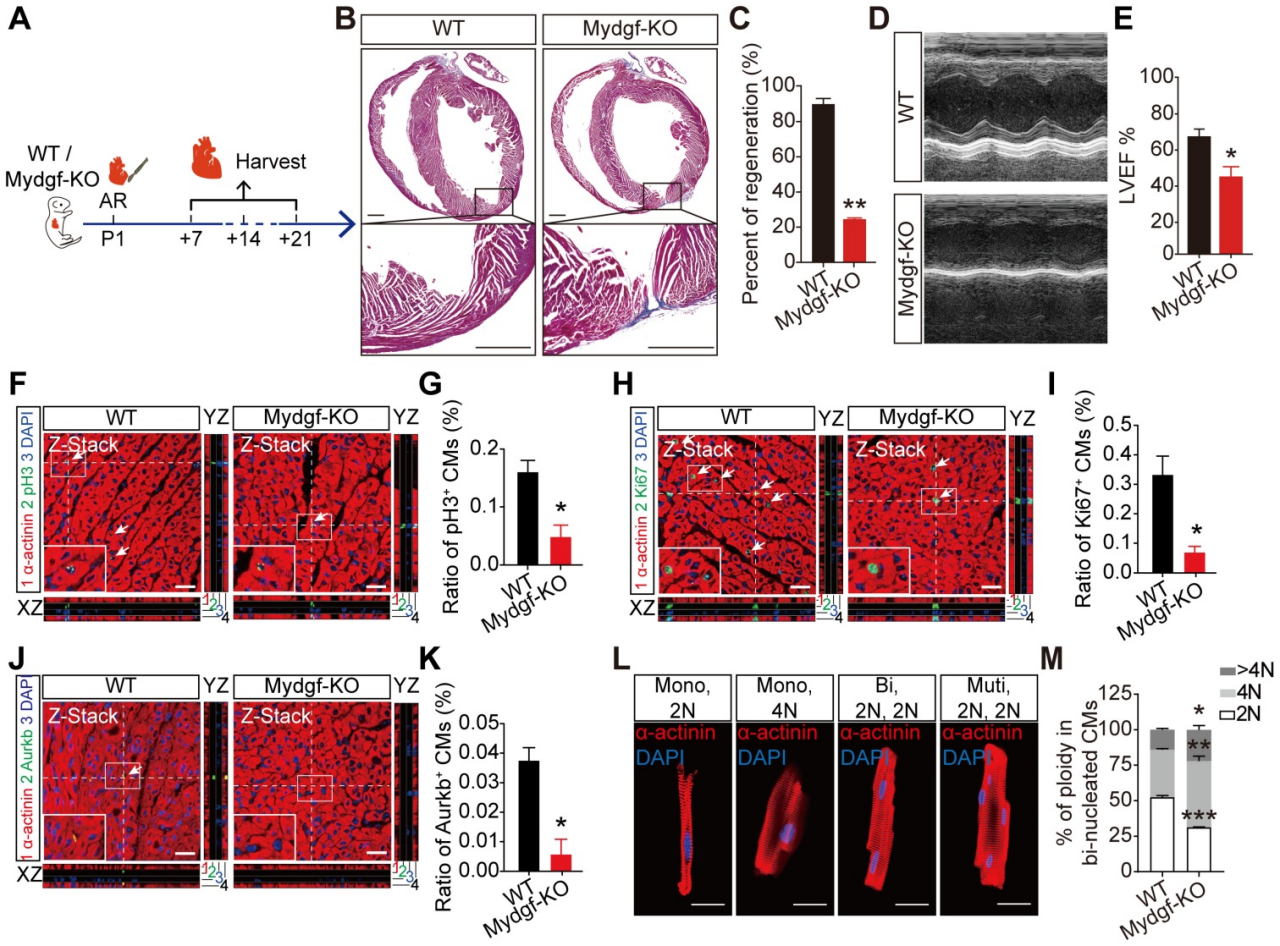
** Mydgf is necessary for heart regeneration and cardiomyocyte proliferation.** (**A and B**) Masson's staining showed heart regeneration in *Mydgf-KO* mice after apical resection (AR) injury relative to wild-type (WT) at 21 days post resection (dpr). Statistical analysis revealed that heart regeneration was reduced in *Mydgf-KO* mice (n = 15 for *Mydgf-KO* mice and n = 10 for WT mice). Scale bars, 500 µm. (**C**) Representative images of echocardiography analysis in *Mydgf-KO* and WT mice at 21 dpr. (**D**) Echocardiography analysis of left ventricular ejection fraction (LVEF) in *Mydgf-KO* and WT mice at 21 dpr (n = 10 for *Mydgf-KO* mice and n = 9 for WT mice). *P < 0.05 and **P < 0.01 compared to WT by Student's *t*-test (B and D). (**E**) Schematic diagram showed the experimental design for F-K. (**F and G**) Immunostaining illustrated proliferative (pH3^+^, green, white arrows) cardiomyocytes (CMs) were decreased in *Mydgf-KO* mice relative to wild-type (WT) at 7 days post resection (dpr) (n = 3 per group). Scale bars, 20 µm. (**H and I**) Immunostaining illustrated proliferative (Ki67^+^, green, white arrows) CMs were decreased in *Mydgf-KO* mice relative to WT at 7 dpr (n = 3 per group). (**J and K**) Immunostaining illustrated proliferative (Aurkb^+^, green, white arrows) CMs were decreased in *Mydgf-KO* mice relative to WT at 7 dpr (n = 3 per group). *P < 0.05 and **P < 0.01 compared to WT by Student's *t*-test (G, I and K). (**L and M**) Example photomicrographs of isolated CMs from *Mydgf-KO* mice heart and quantification of ploidy at 14 dpr (n = 6 per group). *P < 0.05, **P < 0.01 and ***P < 0.001 compared to WT by two-way ANOVA with Bonferroni's multiple comparisons test (M). Scale bars, 20 µm. Values were presented as the mean ± S.E.M.

We performed AR on P1 *Mydgf-KO* mice and evaluated heart regeneration. Masson's staining analysis revealed a remarkably larger fibrosis and a significant decrease of regenerative capacity at 21 dpr in *Mydgf-KO* mice compared with WT littermates (n = 15 for *Mydgf-KO* mice and n = 10 for WT littermates) (Figure 2A-B). Echocardiography displayed the worse heart function in* Mydgf-KO* mice at 21 dpr relative to WT littermates (n = 10 for *Mydgf-KO* mice and n = 9 for WT littermates) (Figure 2C-D), suggesting Mydgf is required for heart regeneration. We analyzed the CM size *via* Wheat germ agglutinin (WGA) straining and found no difference between *Mydgf-KO* mice and WT littermates at 21 dpr (Supplementary Figure 2D-E).

The central feature of heart regenerative response is the activation of CM proliferation following injury 24. We performed AR operation on P1* Mydgf-KO* mice and detected CM proliferation by immunofluorescence staining of mitosis markers phosphorylated Histone 3 (pH3), Ki67, and Aurora B (Aurkb) respectively with CM marker anti-sarcomeric alpha actinin (α-actinin) at 7 dpr (n = 3 per group) (Figure 2E). We found that Mydgf deficiency was harmful for CM proliferation after AR injury (Figure 2F-K) without the disturbance of EC and fibroblast (FB) proliferation (Supplementary Figure 2F-I), indicating Mydgf is necessary for CM proliferation during neonatal heart regeneration.

We determined CM ploidy with confocal microscopy, which enabled quantification of DNA content in individual CM nuclei (Figure 2L). Taking non-CMs as reference for diploid (2N) nuclei, *Mydgf-KO* CM nuclei showed a 40% decrease of 2N nuclei and a 30% increase of polyploid (4N and > 4N) in binucleated CMs at 14 dpr (n = 6 per group) (Figure 2M), indicating that Mydgf deficiency reduces karyokinesis, and thereby increases ploidy.

### Mydgf is sufficient for CM proliferation and heart regeneration

**Figure 3:**
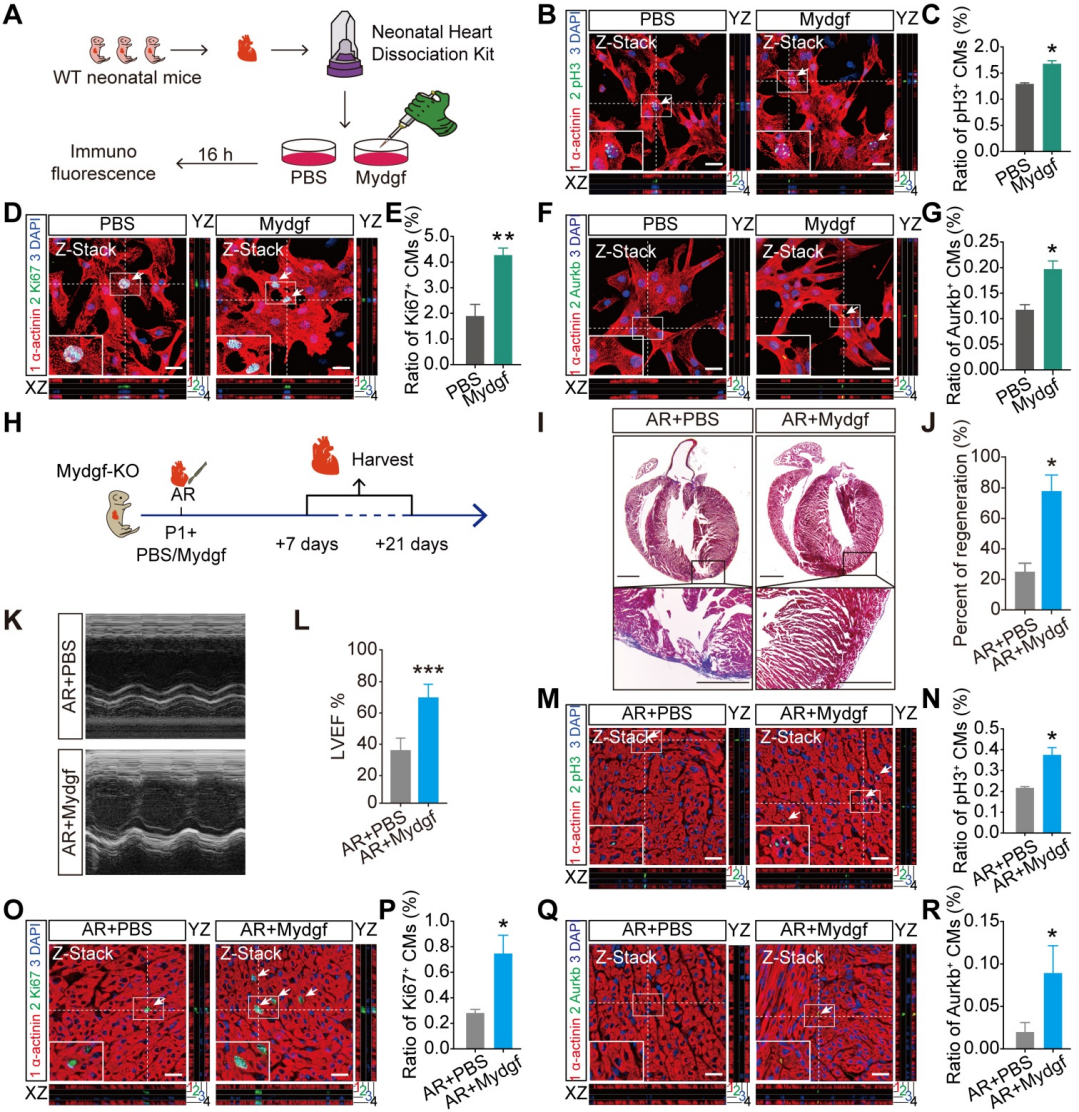
** Mydgf is sufficient for cardiomyocyte proliferation and heart regeneration.** (**A**) Schematic diagram showed the experimental design for B-G. (**B and C**) Immunostaining illustrated proliferative (pH3^+^, green, white arrows) primary cardiomyocytes (CMs) were increased in Mydgf-treated group relative to PBS-treated group after 16 hours (n = 3 per group). (**D and E**) Immunostaining illustrated proliferative (Ki67^+^, green, white arrows) primary CMs were increased in Mydgf-treated group relative to PBS-treated group after 16 hours (n = 3 per group). (**F and G**) Immunostaining illustrated proliferative (Aurkb^+^, green, white arrows) primary CMs were increased in Mydgf-treated group relative to PBS-treated group after 16 hours (n = 3 per group). *P < 0.05 and **P < 0.01 compared to PBS by Student's *t*-test (C, E and G). (**H**) Schematic diagram showed the experimental design for I-R. (**I and J**) Masson's staining showed heart regeneration in *Mydgf-KO* mice treated with Mydgf relative to PBS at 21 days post resection (dpr). Statistical analysis revealed that heart regeneration was induced in Mydgf-treated mice (n = 20 for Mydgf-treated mice and n = 15 for PBS-treated mice). Scale bars, 500 µm. (**K**) Representative images of echocardiography analysis in *Mydgf-KO* mice treated with Mydgf relative to PBS at 21 dpr. (**L**) Echocardiography analysis of left ventricular ejection fraction (LVEF) in *Mydgf-KO* mice treated with Mydgf relative to PBS at 21 dpr (n = 10 for Mydgf-treated mice and n = 9 for PBS-treated mice). *P < 0.05 and ***P < 0.001 compared to PBS-treated mice by Student's *t*-test (J and L). (**M and N**) Immunostaining illustrated proliferative (pH3^+^, green, white arrows) CMs were increased in *Mydgf-KO* neonatal mice treated with Mydgf relative to PBS at 7 dpr (n = 3 per group). (**O and P**) Immunostaining illustrated proliferative (Ki67^+^, green, white arrows) CMs were increased in *Mydgf-KO* neonatal mice treated with Mydgf relative to PBS at 7 days post resection (dpr) (n = 3 per group). (**Q and R**) Immunostaining illustrated proliferative (Aurkb^+^, green, white arrows) CMs were increased in *Mydgf-KO* neonatal mice treated with Mydgf relative to PBS at 7 dpr (n = 3 per group). Scale bars, 20 µm. *P < 0.05 compared to AR+PBS by Student's *t*-test (N, P and R). Values were presented as the mean ± S.E.M.

To investigate whether Mydgf could stimulate CM proliferation, we isolated neonatal primary CMs from WT mice, and treated with Mydgf recombinant protein (n = 3 per group) (Figure 3A). Immunofluorescence staining showed that pH3^+^, Ki67^+^ and Aurkb^+^ CMs were significantly increased after 16 hours in Mydgf-treated CMs compared with PBS group (Figure 3B-G), suggesting that Mydgf promotes CM proliferation* in vitro*.

We microinjected Mydgf recombinant protein (1 µg/mouse) into *Mydgf-KO* P1 mouse myocardium after AR injury (Figure 3H). Masson's staining and echocardiography analysis demonstrated that the *Mydgf-KO* mice treated with Mydgf showed completed heart regeneration (n = 20 for Mydgf-treated mice and n = 15 for PBS-treated mice) (Figure 3I-J) and enhanced systolic dysfunction (n = 10 for Mydgf-treated mice and n = 9 for PBS-treated mice) (Figure 3K-L) at 21 dpr. Furthermore, we detected CM proliferation at 7 dpr by immunofluorescence staining (n = 3 per group) and found that CM proliferation was enhanced significantly at 7 dpr (Figure 3M-R). These results indicate that Mydgf is competent to improve CM proliferation* in vivo* and improve neonatal heart regeneration after cardiac injury.

### Mydgf controls CM proliferation through c-Myc/FoxM1 pathway

**Figure 4:**
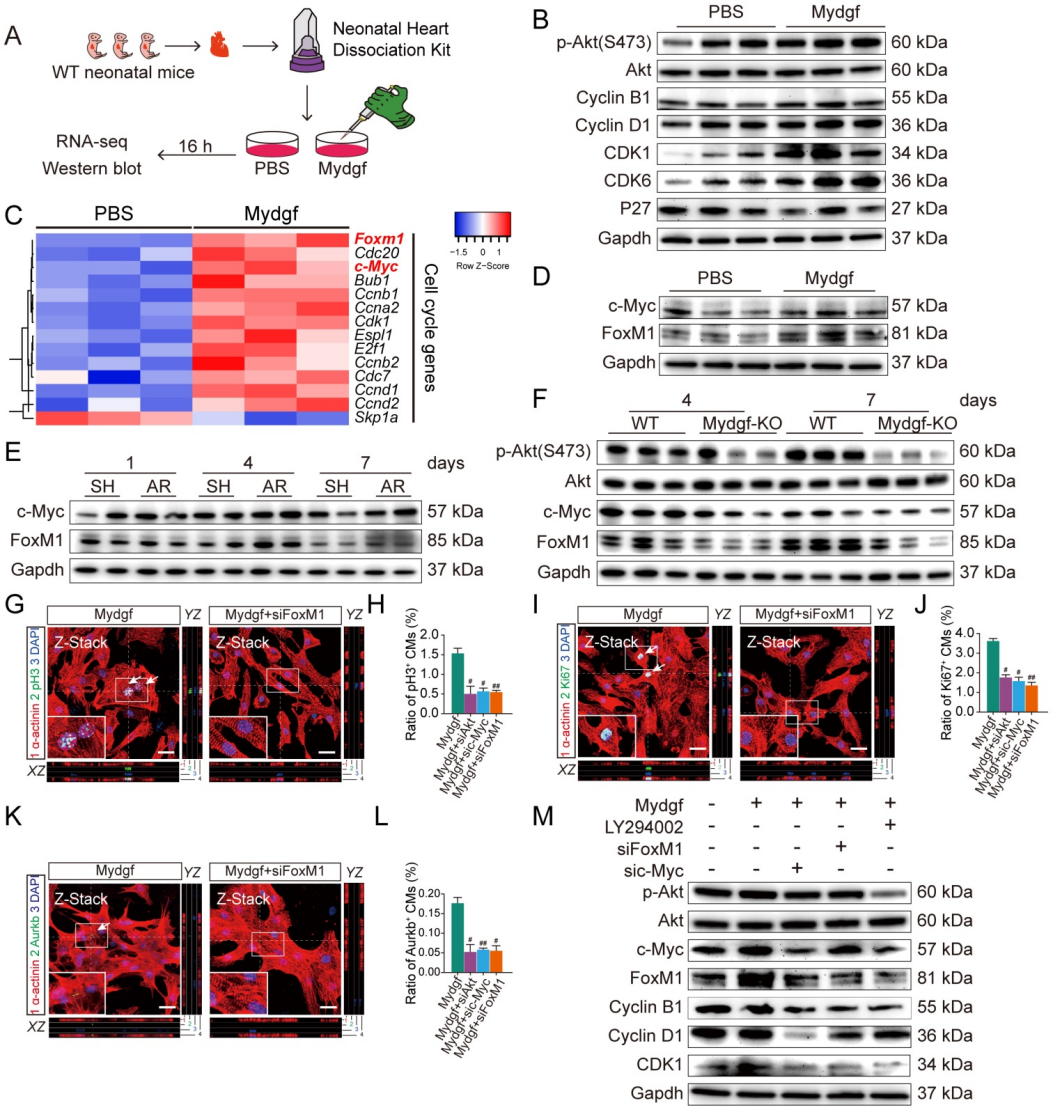
** Mydgf controls cardiomyocyte proliferation through c-Myc/FoxM1 pathway.** (**A**) Schematic diagram showed the experimental design for B-F. (**B**) Western blot of p-Akt and cell cycle relative protein in PBS and Mydgf-treated cardiomyocytes (CMs). (**C**) Heat map of genes under cell cycle regulated networks. (**D**) Western blot of c-Myc and FoxM1 in primary CMs treated with PBS and Mydgf. (**E**) Western blot of c-Myc and FoxM1 in wild-type (WT) mouse hearts harvested at 1, 4, 7 dpr. (**F**) Western blot of p-Akt, c-Myc and FoxM1 of hearts harvested at 4, 7 days post resection (dpr) in wild-type (WT) or *Mydgf-KO* mouse hearts. (**G and H**) Immunostaining illustrated proliferative (pH3^+^, green, white arrows) primary CMs were decreased treated with siRNA-Akt, c-Myc and FoxM1 respectively prior to Mydgf treatment relative to siRNA-NC treatment group after 48 hours (n = 3 per group). (**I and L**) Immunostaining illustrated proliferative (Ki67^+^, green, white arrows) primary CMs were decreased treated with siRNA-Akt, c-Myc and FoxM1 respectively prior to Mydgf treatment relative to siRNA-NC treatment group after 48 hours (n = 3 per group). (**K and L**) Immunostaining illustrated proliferative (Aurkb^+^, green, white arrows) primary CMs were decreased treated with siRNA-Akt, c-Myc and FoxM1 respectively prior to Mydgf treatment relative to siRNA-NC treatment group after 48 hours (n = 3 per group). ^#^*P* < 0.05 and ^##^*P* < 0.01 compared to Mydgf treatment by Student's *t*-test (**H, J and L**). (**M**) Western blot of p-Akt, c-Myc, FoxM1, Cyclin B1, Cyclin D1 and CDK1 in primary CMs transfected with different treatment. Values were presented as the mean ± S.E.M.

Employing RNA sequencing (RNA-Seq), we explored the gene expression profile in Mydgf-treated neonatal mouse primary (n = 3 per group) (Figure 4A) and found that a total of 1,005 genes were differentially expressed after 16 hours Mydgf treatment, including 298 downregulated genes and 707 upregulated genes. Kyoto encyclopedia of genes and genomes (KEGG) analysis of the upregulated genes revealed significant enrichment in the cell cycle and phosphatidylinositol 3-kinase (PI3K)-Akt signaling pathways (Supplementary Figure 3A). Western blot revealed that the cell cycle-related proteins Cyclin B1, Cyclin D1, CDK1, and CDK6, as well as the phosphorylation (p) of Akt (Ser473) were obviously increased in the Mydgf-treated CMs (n = 3 per group) (Figure 4B, Supplementary Figure 3B). However, the phosphorylation of STAT3 on S727 and Y705 in primary CMs was unchanged after Mydgf treatment (n = 3 per group) (Supplementary Figure 3C-D). The heat map of cell cycle genes (fold change > 1 in log 2 scale and adjusted *P* < 0.05) between Mydgf- and PBS-treated CMs was shown in Figure 4C. We found that Forkhead box M1 (FoxM1) and Myc were significantly increased in Mydgf-treated CMs and verified by western blot (n = 3 per group) (Figure 4D, Supplementary Figure 3E).

We found that both c-Myc and FoxM1 were increased in WT mouse myocardium at 4 and 7 dpr compared with sham-operation WT mice (n = 4 per group, two hearts as a sample) (Figure 4E, Supplementary Figure 3F-G). Moreover, we also performed AR operation on neonatal *Mydgf-KO* and WT mice. Western blot showed that Mydgf deficiency decreased the expression of p-Akt, c-Myc, and FoxM1 at 4 dpr and 7 dpr relative to WT littermates (n = 4 per group, two hearts as a sample) (Figure 4F, Supplementary Figure 3H), suggesting that c-Myc and FoxM1 are indispensable in heart regeneration.

Both FoxM1 and c-Myc have been identified as the pro-proliferative factors 25, 26. To investigate whether the c-Myc/FoxM1 mediated Mydgf-induced CM proliferation, we treated primary CMs with small interfering RNA (siRNA)-Akt, c-Myc, or FoxM1 respectively prior to Mydgf treatment. We found that Mydgf-induced CM proliferation was remarkably inhibited by knockdown of Akt, c-Myc, or FoxM1 (Figure 4G-L), respectively, with the blockage of cell cycle-related gene expression, including Ki67, Cyclin B1, and CDK1 (n = 6 per group) (Supplementary Figure 3I). Western blot showed that Mydgf-induced p-Akt, Cyclin B1, Cyclin D1, and CDK1 activation were suspended by Akt inhibitor LY294002, siRNA-c-Myc, and siRNA-FoxM1(n = 3 per group) (Figure 4M, Supplementary Figure 3K). We also found that Akt knockdown suspended both c-Myc and FoxM1 expression, and c-Myc knockdown blocked FoxM1 expression, while FoxM1 knockdown had no effect on Akt and c-Myc (n = 6 per group) (Figure 4M, Supplementary Figure 3J-K), indicating that Akt mediated Mydgf-induced CM proliferation *via* c-Myc/FoxM1 pathway.

### Mydgf improves heart regeneration in adult mice

To validate the function of Mydgf in adult mice, we established myocardial infarction (MI) mouse model by the ligation of the left anterior descending (LAD) coronary artery in adult *Mydgf-KO* mice (Supplementary Figure 4A). Histological analysis revealed that *Mydgf-KO* mice had a poor capacity for cardiac repair (n = 25 for *Mydgf-KO* mice and n = 13 for WT mice) (Supplementary Figure 4B-4C). Echocardiography showed a conspicuous decrease in cardiac function (n = 19 for *Mydgf-KO* mice and n = 14 for WT mice) (Supplementary Figure 4D-4E) at 21 days post infarction (dpi). Moreover, the survival rate of *Mydgf-KO* mice was significantly lower than WT littermates (Supplementary Figure 4F) (n = 25 for *Mydgf-KO* mice and n = 29 for WT mice), suggesting that Mydgf is necessary for cardiac repair in adult mice.

**Figure 5:**
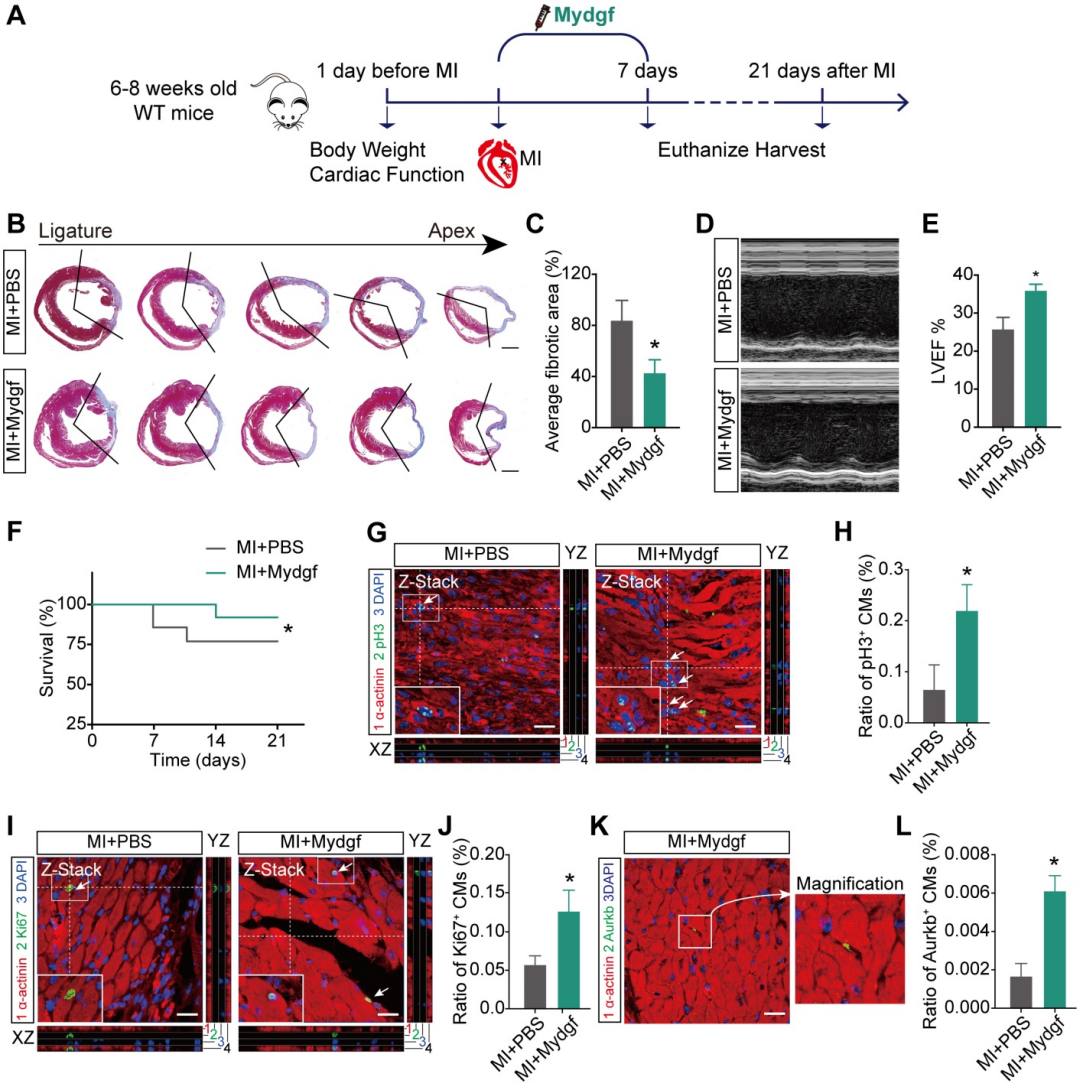
** Mydgf promotes heart regeneration in adult mice.** (**A**) Schematic diagram showed the experimental design for B-L. (**B and C**) Masson's staining elucidated the infarcted area in wild-type (WT) adult mice treated with PBS/Mydgf at 21 days post infarction (dpi). Statistical analysis of fibrotic area showed the infarcted size was significantly smaller in adult WT mice treated with Mydgf at 21 dpi relative to PBS (n = 25 for Mydgf-treated mice and n = 13 for PBS-treated mice). Scale bars, 500 µm. (**D**) Representative images of echocardiography analysis in adult WT mice treated with PBS/Mydgf at 21 dpi. (**E**) Echocardiography analysis of left ventricular ejection fraction (LVEF) in adult WT mice treated with PBS/Mydgf at 21 dpi (n = 19 for Mydgf-treated mice and n = 13 for PBS-treated mice). *P < 0.05 and **P < 0.01 compared to WT by Student's *t*-test (C and E). (**F**) Cumulative survival after MI in WT mice treated with 25 PBS and 20 Mydgf. *P < 0.05 compared to PBS-treated group by log-rank test. (**G and H**) Immunostaining illustrated proliferative (pH3^+^, green, white arrows) cardiomyocytes (CMs) were increased in WT adult mice treated with Mydgf relative to PBS at 7 dpi (n = 3 per group). (**I and J**) Immunostaining illustrated proliferative (Ki67^+^, green, white arrows) CMs were increased in WT adult mice treated with Mydgf relative to PBS at 7 dpi (n = 3 per group). (**K and L**) Immunostaining illustrated proliferative (Aurkb^+^, green, white arrows) CMs were increased in WT adult mice treated with Mydgf relative to PBS at 7 dpi (n = 3 per group). Scale bars, 20 µm. *P < 0.05 and **P < 0.01 compared to PBS-treated group by Student's *t*-test (H, J and L). Values were presented as the mean ± S.E.M.

We also microinjected Mydgf recombinant protein (5 µg/mouse) into WT adult mice hearts after MI followed by a continuous tail vein injection for 7 days (10 µg/d) (Figure 5A). Masson's staining and echocardiography analysis showed that Mydgf-treated hearts showed reduced infarct size (n = 25 for Mydgf-treated mice and n = 13 for PBS-treated mice) (Figure 5B-C) and developed less pronounced systolic dysfunction (n = 19 for Mydgf-treated mice and n = 13 for PBS-treated mice) (Figure 5D-E) at 21 dpi. The Mydgf-treated mice were associated with a marked survival benefit (n = 25 for Mydgf-treated mice and n = 20 for PBS-treated mice) (Figure 5F). Immunofluorescence staining showed that CM proliferation was increased in Mydgf-treated mice at 7 dpi (Figure 5G-L). These results indicate that Mydgf promotes adult mouse heart regeneration and CM proliferation after MI, suggesting that Mydgf might be an effective therapeutic target for reversing cardiac remodeling and guarding against heart failure.

## Discussion

Mydgf has been considered as a cardiac protective factor and mainly secreted by bone marrow-derived mononuclear MΦs 18. Here we found that Mydgf was secreted mainly by ECs rather than MΦs after neonatal AR injury. We also demonstrated that Mydgf could promote CM proliferation after neonatal heart injury and adult MI, providing a new insight to understand the function of Mydgf in heart regeneration. FoxM1 has been reported as a transcription factor regulating CM proliferation 26. Employing RNA-Seq and functional experiments, we identified that the c-Myc/FoxM1 pathway mediated Mydgf-induced CM proliferation. These findings elucidate the mechanism of Mydgf in promoting heart regeneration after cardiac injury.

Mydgf has been reported to play a crucial role during cardiac repair, such as promoting angiogenesis and inhibiting CM apoptosis 18, 19, 27. Our results display that the proliferation of ECs and FBs was not significantly induced by Mydgf after neonatal cardiac injury, suggesting that Mydgf-induced CM proliferation is the major factor for neonatal heart regeneration. It has been reported that Mydgf promotes EC proliferation through phosphorylation of STAT3 on S727 and stimulates STAT3 transcriptional activity 18, 19. Our results show that STAT3 is not be activated in Mydgf-treated CMs, indicating that STAT3 signaling is not involved in Mydgf-indcued CM proliferation. We also found that Mydgf deficiency reduced karyokinesis, and thereby increased ploidy, which is coincided with reported data 17.

In summary, our study demonstrates the role of Mydgf in neonatal and adult heart regeneration, which provides an effective therapeutic target for perfect against heart failure after cardiac injury. We also identify a novel pathway involved in CM proliferation, c-Myc/FoxM1, underlying Mydgf-induced heart regeneration. These findings broaden the understanding of the role of Mydgf and provide new insights into mechanisms for heart regeneration.

## Supplementary Material

Supplementary figures.

## Abbreviations

Mydgf: myeloid-derived growth factor
CM: cardiomyocyte
WT: wild-type
P: postnatal day
qRT-PCR: quantitative real-time Polymerase Chain Reaction
AR: apical resection
MI: myocardial infarction
dpr: days post resection
dpi: days post infarction
*Mydgf-KO*: global Mydgf knockout
LVEF: left ventricular ejection fraction
LAD: left anterior descending
pH3: phosphorylated Histone 3
Aurkb: aurora kinase B
EC: endothelial cell
MΦ: macrophage
FB: fibroblast
KEGG: kyoto encyclopedia of genes and genomes
RNA-Seq: RNA sequencing
PI3K: phosphatidylinositol 3-kinase
FoxM1: Forkhead box M1
siRNA: small interfering RNA
NC: negative control

## References

[1] Ponnusamy M, Liu F, Zhang YH, Li RB, Zhai M, Liu F (2019). Long Noncoding RNA CPR (Cardiomyocyte Proliferation Regulator) Regulates Cardiomyocyte Proliferation and Cardiac Repair. Circulation.

[2] Song SY, Yoo J, Go S, Hong J, Sohn HS, Lee JR (2019). Cardiac-mimetic cell-culture system for direct cardiac reprogramming. Theranostics.

[3] Zhou Q, Pan LL, Xue R, Ni G, Duan Y, Bai Y (2020). The anti-microbial peptide LL-37/CRAMP levels are associated with acute heart failure and can attenuate cardiac dysfunction in multiple preclinical models of heart failure. Theranostics.

[4] Wang L, Zhang F, Duan F, Huang R, Chen X, Ming J (2020). Homozygous MESP1 knock-in reporter hESCs facilitated cardiovascular cell differentiation and myocardial infarction repair. Theranostics.

[5] Feng J, Li Y, Nie Y (2018). Non-Cardiomyocytes in Heart Regeneration. Curr Drug Targets.

[6] Li H, Bao M, Nie Y (2020). Extracellular matrix-based biomaterials for cardiac regeneration and repair. Heart Fail Rev.

[7] Li Y, Li H, Pei J, Hu S, Nie Y (2020). Transplantation of murine neonatal cardiac macrophage improves adult cardiac repair. Cell Mol Immunol.

[8] Lam NT, Sadek HA (2018). Neonatal Heart Regeneration. Circulation.

[9] Porrello ER, Mahmoud AI, Simpson E, Hill JA, Richardson JA, Olson EN (2011). Transient regenerative potential of the neonatal mouse heart. Science.

[10] Vandergriff A, Huang K, Shen D, Hu S, Hensley MT, Caranasos TG (2018). Targeting regenerative exosomes to myocardial infarction using cardiac homing peptide. Theranostics.

[11] Alkass K, Panula J, Westman M, Wu TD, Guerquin-Kern JL, Bergmann O (2015). No Evidence for Cardiomyocyte Number Expansion in Preadolescent Mice. Cell.

[12] Li J, Yang KY, Tam RCY, Chan VW, Lan HY, Hori S (2019). Regulatory T-cells regulate neonatal heart regeneration by potentiating cardiomyocyte proliferation in a paracrine manner. Theranostics.

[13] Hao K, Lei W, Wu H, Wu J, Yang Z, Yan S (2019). LncRNA-Safe contributes to cardiac fibrosis through Safe-Sfrp2-HuR complex in mouse myocardial infarction. Theranostics.

[14] Payumo AY, Huang GN (2020). Lamin B2, Guardian of Cardiomyocyte Nuclear Division. Dev Cell.

[15] Patterson M, Barske L, Van Handel B, Rau CD, Gan P, Sharma A (2017). Frequency of mononuclear diploid cardiomyocytes underlies natural variation in heart regeneration. Nat Genet.

[16] Hirose K, Payumo AY, Cutie S, Hoang A, Zhang H, Guyot R (2019). Evidence for hormonal control of heart regenerative capacity during endothermy acquisition. Science.

[17] Han L, Choudhury S, Mich-Basso JD, Ammanamanchi N, Ganapathy B, Suresh S (2020). Lamin B2 Levels Regulate Polyploidization of Cardiomyocyte Nuclei and Myocardial Regeneration. Dev Cell.

[18] Korf-Klingebiel M, Reboll MR, Klede S, Brod T, Pich A, Polten F (2015). Myeloid-derived growth factor (C19orf10) mediates cardiac repair following myocardial infarction. Nat Med.

[19] Zhao L, Feng S, Wang S, Fan M, Jin W, Li X (2020). Production of bioactive recombinant human myeloid-derived growth factor in Escherichia coli and its mechanism on vascular endothelial cell proliferation. J Cell Mol Med.

[20] Mahmoud AI, Porrello ER, Kimura W, Olson EN, Sadek HA (2014). Surgical models for cardiac regeneration in neonatal mice. Nat Protoc.

[21] Li Y, Feng J, Li Y, Hu S, Nie Y (2020). Achieving stable myocardial regeneration after apical resection in neonatal mice. J Cell Mol Med.

[22] Han C, Nie Y, Lian H, Liu R, He F, Huang H (2015). Acute inflammation stimulates a regenerative response in the neonatal mouse heart. Cell Res.

[23] Yue Z, Chen J, Lian H, Pei J, Li Y, Chen X (2019). PDGFR-beta Signaling Regulates Cardiomyocyte Proliferation and Myocardial Regeneration. Cell Rep.

[24] Cahill TJ, Choudhury RP, Riley PR (2017). Heart regeneration and repair after myocardial infarction: translational opportunities for novel therapeutics. Nat Rev Drug Discov.

[25] Ho C, Wang C, Mattu S, Destefanis G, Ladu S, Delogu S (2012). AKT (v-akt murine thymoma viral oncogene homolog 1) and N-Ras (neuroblastoma ras viral oncogene homolog) coactivation in the mouse liver promotes rapid carcinogenesis by way of mTOR (mammalian target of rapamycin complex 1), FOXM1 (forkhead box M1)/SKP2, and c-Myc pathways. Hepatology.

[26] Sengupta A, Yutzey KE, Kalinichenko VV (2013). FoxO1 and FoxM1 transcription factors have antagonistic functions in neonatal cardiomyocyte cell-cycle withdrawal and IGF1 gene regulation. Circ Res.

[27] Polten F, Reboll MR, Widera C, Kempf T, Bethmann K, Gupta P (2019). Plasma Concentrations of Myeloid-Derived Growth Factor in Healthy Individuals and Patients with Acute Myocardial Infarction as Assessed by Multiple Reaction Monitoring-Mass Spectrometry. Anal Chem.

