# Supplementary material for: Mydgf promotes Cardiomyocyte proliferation and Neonatal Heart regeneration
Source: Theranostics. 2020 Jul 11;10(20):9100–12. doi: 10.7150/thno.44281 (PMC7415811; doi:10.7150/thno.44281)
Supplement: Supplementary file 1 — Supplementary figures. [file thnov10p9100s1.pdf]

Supplemental Figure

Supplemental Figure 1.

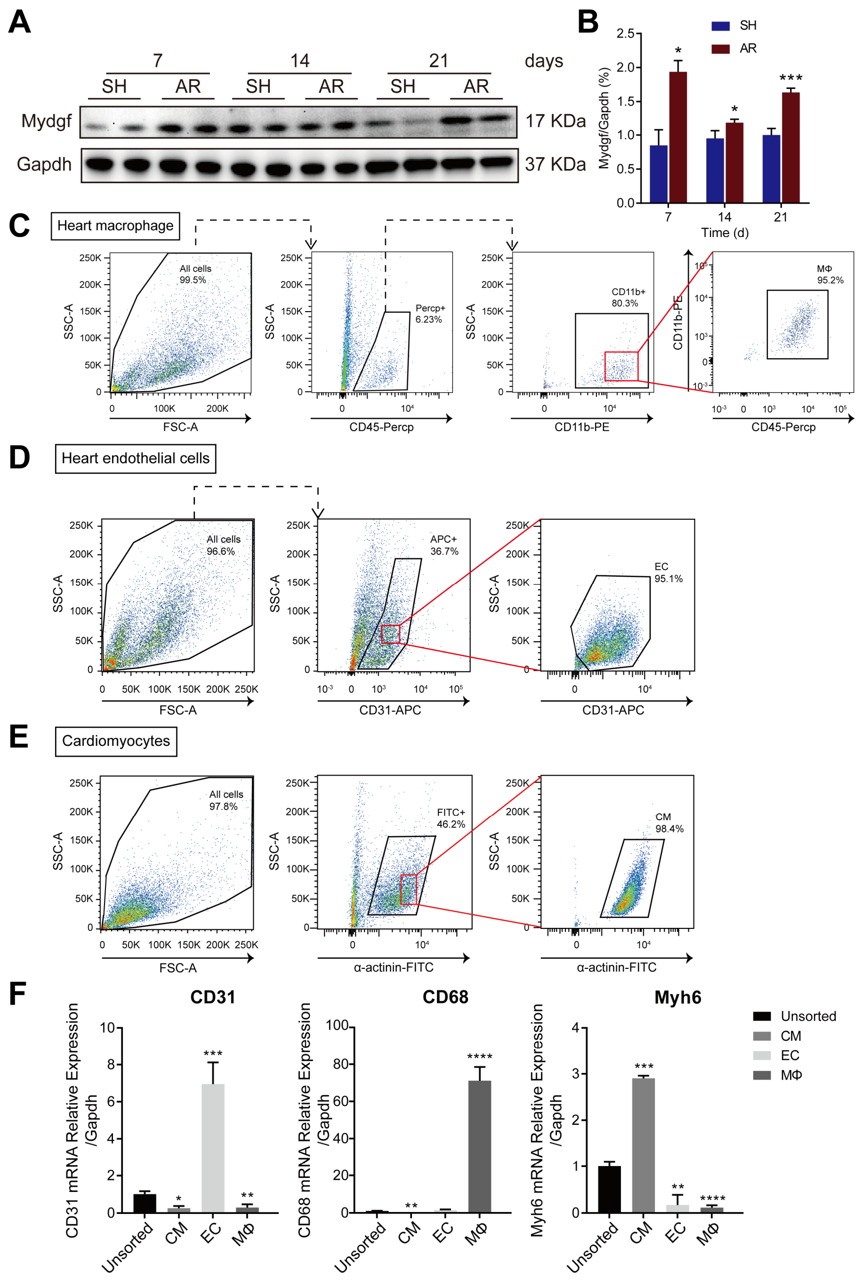

Supplemental Figure 1. Mydgf is upregulated in injured neonatal mouse hearts

(A-B) Western blot analysis of Mydgf in wild-type mouse heart harvested at 7, 14, 21 days post resection (dpr). Statistical analysis revealed that the expression of Mydgf was upregulated at 7, 14 and 21 dpr (n = 3 per group). **P* < 0.05 and ****P* < 0.001 compared to sham (SH) at the corresponding time-point by Student's *t*-test (B). (C-E) The gating

strategy of heart macrophages (MΦs), endothelial cells (ECs) and cardiomyocytes (CMs) by Flow cytometry. (F) qRT-PCR analysis of three cell populations (ECs, MΦs and CMs) for CD31 (an EC marker), CD68 (a MΦ marker) and Myh6 (a CM marker) (n = 3 per group). $^{*}P < 0.05$, $^{**}P < 0.01$, $^{***}P < 0.001$ and $^{****}P < 0.0001$ compared to Unsorted by Student's *t*-test. Values were presented as the mean ± S.E.M.

Supplemental Figure 2.

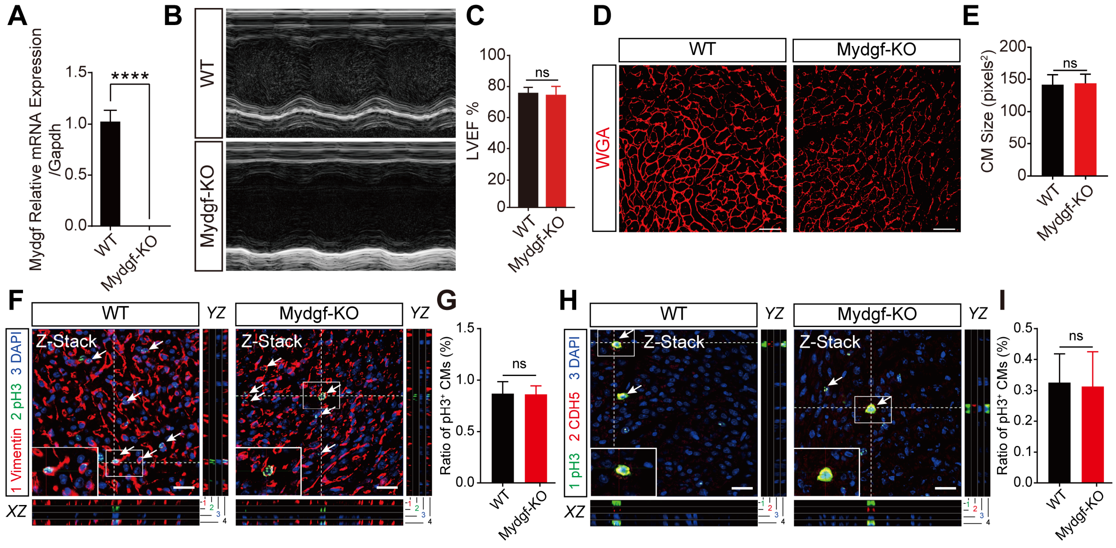

Supplemental Figure 2. Mydgf deficiency has no effect on cardiomyocyte size

(A) qRT-PCR analysis of Mydgf expression in *Mydgf-KO* and wild-type (WT) mouse hearts (n = 3 per group). *****P* < 0.0001 compared to WT by Student's *t*-test. (B) Representative images of echocardiography analysis in *Mydgf-KO* and WT mice at postnatal day 21 (P21). (C) Echocardiography analysis of left ventricle ejection fraction (LVEF) in *Mydgf-KO* and WT mice at P21 (n = 9 for *Mydgf-KO* and n = 6 for WT). ns, no statistical significance. (D and E) Immunostaining showed the size (WGA, red) of cardiomyocytes (CMs) in *Mydgf-KO* mice and WT controls at 21 days post resection (dpr). Scale bars, 20 μm. Statistical analysis revealed that there is no size difference in CMs between *Mydgf-KO* mice and WT (n = 6 per group). ns, no statistical significance. (F-I) Fibroblast (Vimentin^{+}, red) and endothelial cell (CDH5^{+}, red) proliferation was showed by pH3 (green) immunostaining in *Mydgf-KO* and WT mice at 7 dpr performed at P1. Statistical analysis revealed that there are no obvious differences in the proliferation of fibroblasts and endothelial cells between *Mydgf-KO* mice and WT at 7

dpr (n = 3 per group). Scale bars, 20 μm. ns, no statistical significance. Values were presented as the mean ± S.E.M.

Supplemental Figure 3.

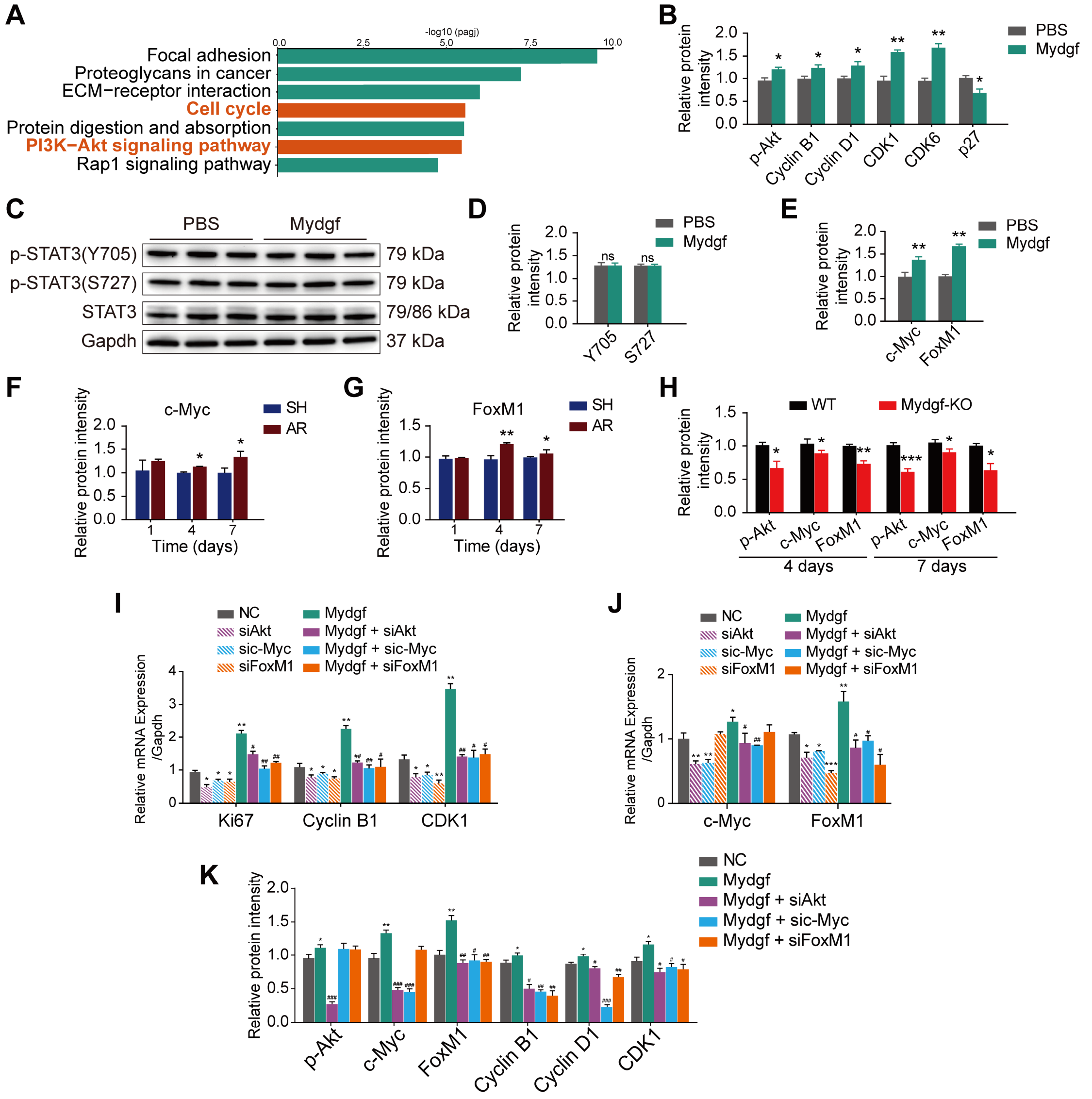

Supplemental Figure 3. Mydgf induces PI3K, c-Myc and FoxM1 activation in cardiomyocytes

(A) Kyoto encyclopedia of genes and genomes (KEGG) analysis of the upregulated gene significantly enriched terms after Mydgf treatment by RNA sequencing. (B) Statistical analysis revealed that the expression of p-Akt and cell cycle relative protein in primary cardiomyocytes (CMs) was upregulated after Mydgf treatment (n = 3 per group) (Related to Figure 4B). *$P < 0.05$ and **$P < 0.01$ compared to PBS by Student's

t-test. (C and D) Western blot of p-STAT3 (Y705) and p-STAT3 (Y727) in primary CMs treated with PBS and Mydgf. Statistical analysis revealed that the expression of p-STAT3 (Y705) and p-STAT3 (Y727) was unchanged after Mydgf treatment (n = 3 per group). ns, no statistical significance. (E) Statistical analysis revealed that the expression of c-Myc and FoxM1 in primary CMs was upregulated after Mydgf treatment (n = 3 per group) (Related to Figure 4D). $^{**}P < 0.01$ compared to PBS by Student's *t*-test. (F and G) Statistical analysis revealed that the expression of c-Myc and FoxM1 was increased in wild-type (WT) mouse hearts at 4 and 7 days post resection (dpr) (n = 4 per group, two hearts as a sample) (Related to Figure 4E). $^{*}P < 0.05$ and $^{**}P < 0.01$ compared to SH at corresponding time-point by Student's *t*-test. (H) Statistical analysis revealed that the expression of p-Akt, c-Myc and FoxM1 was decreased in *Mydgf-KO* mouse hearts at 4 and 7 dpr compared to WT mice (n = 4 per group, two hearts as a sample) (Related to Figure 4F). $^{*}P < 0.05$, $^{**}P < 0.01$, $^{***}P < 0.001$ compared to WT by Student's *t*-test. (I) qRT-PCR analysis of *Ki67*, *Cyclin B1*, and *CDK1* in primary CMs transfected with different treatments. Statistical analysis revealed that knockdowns decreased the expression of the cell cycle-related genes (n = 6 per group). (J) qRT-PCR analysis of *c-Myc* and *FoxM1* in primary CMs transfected with different treatment. Statistical analysis revealed that knockdown of Akt reduced the expression of c-Myc and FoxM1, and FoxM1 expression was also reduced by siRNA-c-Myc (n = 6 per group). (K) Statistical analysis revealed that the knockdowns of Akt, c-Myc and FoxM1 in primary CMs decreased the expression of the cell cycle-related protein, p-Akt, c-Myc and FoxM1 (n = 3 per group) (Related to Figure 4M). $^{*}P$

< 0.05, $^{**}P < 0.01$, $^{***}P < 0.001$ compared to NC and $^{\#}P < 0.05$, $^{\#\#}P < 0.01$, $^{\#\#\#}P <$ 0.001 compared to Mydgf treatment by two-way ANOVA with Bonferroni's multiple comparisons test (I, J and K). Values were presented as the mean ± S.E.M.

Supplemental Figure 4.

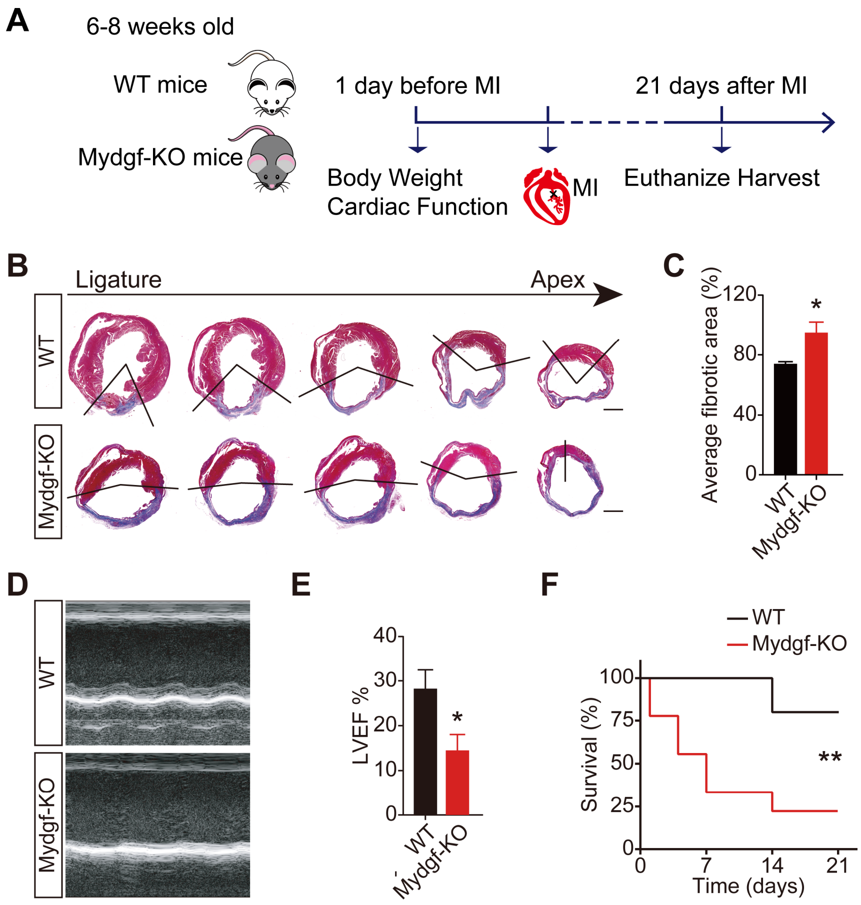

Supplemental Figure 4. Mydgf deficiency impedes cardiac repair after injury in adult mice

(A) Schematic diagram showed the experimental design for B-F. (B and C) Masson's staining elucidated the infarcted area in adult wild-type (WT) and *Mydgf-KO* mice after myocardial infarction (MI) at 21 days post infarction (dpi). Statistical analysis of fibrotic area showed the infarcted size was significantly larger in *Mydgf-KO* mice at 21 dpi relative to WT mice (n = 25 for *Mydgf-KO* mice and n = 13 for WT mice). Scale bars, 500 μm. (D) Representative images of echocardiography analysis in WT and *Mydgf-KO* mice at 21 dpi. (E) Echocardiography analysis of left ventricular ejection fraction (LVEF) in *Mydgf-KO* and WT mice at 21 dpi (n = 19 for *Mydgf-KO* mice and

n = 14 for WT mice). *$P < 0.05$ and **$P < 0.01$ compared to WT by Student's *t*-test (C and E). (F) Cumulative survival after MI in 29 WT and 25 *Mydgf-KO* mice. **$P < 0.01$ compared to WT by log-rank test. Values were presented as the mean ± S.E.M.
